# Analyses of germline variants associated with ovarian cancer survival identify functional candidates at the 1q22 and 19p12 outcome loci

**DOI:** 10.18632/oncotarget.18501

**Published:** 2017-06-15

**Authors:** Dylan M. Glubb, Sharon E. Johnatty, Michael C.J. Quinn, Tracy A. O’Mara, Jonathan P. Tyrer, Bo Gao, Peter A. Fasching, Matthias W. Beckmann, Diether Lambrechts, Ignace Vergote, Digna R. Velez Edwards, Alicia Beeghly-Fadiel, Javier Benitez, Maria J. Garcia, Marc T. Goodman, Pamela J. Thompson, Thilo Dörk, Matthias Dürst, Francesmary Modungo, Kirsten Moysich, Florian Heitz, Andreas du Bois, Jacobus Pfisterer, Peter Hillemanns, On behalf of the AGO Study Group, Beth Y. Karlan, Jenny Lester, Ellen L. Goode, Julie M. Cunningham, Stacey J. Winham, Melissa C. Larson, Bryan M. McCauley, Susanne Krüger Kjær, Allan Jensen, Joellen M. Schildkraut, Andrew Berchuck, Daniel W. Cramer, Kathryn L. Terry, Helga B. Salvesen, Line Bjorge, Penny M. Webb, Peter Grant, Tanja Pejovic, Melissa Moffitt, Claus K. Hogdall, Estrid Hogdall, James Paul, Rosalind Glasspool, Marcus Bernardini, Alicia Tone, David Huntsman, Michelle Woo, AOCS Group, Anna deFazio, Catherine J. Kennedy, Paul D.P. Pharoah, Stuart MacGregor, Georgia Chenevix-Trench

**Affiliations:** ^1^ Department of Genetics and Computational Biology, QIMR Berghofer Medical Research Institute, Brisbane, QLD, Australia; ^2^ Department of Oncology, Centre for Cancer Genetic Epidemiology, University of Cambridge, Strangeways Research Laboratory, Cambridge, UK; ^3^ Crown Princess Mary Cancer Care Centre, Westmead Hospital, Sydney, NSW, Australia; ^4^ Center for Cancer Research, The Westmead Institute for Medical Research, The University of Sydney, Sydney, NSW, Australia; ^5^ University of California at Los Angeles, David Geffen School of Medicine, Department of Medicine, Division of Hematology and Oncology, Los Angeles, CA, USA; ^6^ University Hospital Erlangen, Department of Gynecology and Obstetrics, Friedrich-Alexander-University Erlangen-Nuremberg, Comprehensive Cancer Center Erlangen-EMN, Erlangen, Germany; ^7^ Vesalius Research Center, VIB, Leuven, Belgium; ^8^ Laboratory for Translational Genetics, Department of Oncology, University of Leuven, Leuven, Belgium; ^9^ Division of Gynecologic Oncology, Department of Obstetrics and Gynaecology and Leuven Cancer Institute, University Hospitals Leuven, Leuven, Belgium; ^10^ Vanderbilt Epidemiology Center, Vanderbilt Genetics Institute, Department of Obstetrics and Gynecology, Vanderbilt University School of Medicine, Nashville, TN, USA; ^11^ Division of Epidemiology, Department of Medicine, Vanderbilt Epidemiology Center and Vanderbilt-Ingram Cancer Center, Vanderbilt University School of Medicine, Nashville, TN, USA; ^12^ Human Genetics Group, Spanish National Cancer Centre (CNIO), and Biomedical Network on Rare Diseases (CIBERER), Madrid, Spain; ^13^ Cancer Prevention and Control, Samuel Oschin Comprehensive Cancer Institute, Cedars-Sinai Medical Center, Los Angeles, CA, USA; ^14^ Community and Population Health Research Institute, Department of Biomedical Sciences, Cedars-Sinai Medical Center, Los Angeles, CA, USA; ^15^ Gynaecology Research Unit, Hannover Medical School, Hannover, Germany; ^16^ Department of Gynaecology, University of Jena, Jena, Germany; ^17^ Division of Gynecologic Oncology, Department of Obstetrics, Gynecology and Reproductive Sciences, University of Pittsburgh School of Medicine, Pittsburgh, PA, USA; ^18^ Department of Epidemiology, University of Pittsburgh Graduate School of Public Health, Pittsburgh, PA, USA; ^19^ Ovarian Cancer Center of Excellence, University of Pittsburgh, Pittsburgh, PA, USA; ^20^ Cancer Pathology & Prevention, Division of Cancer Prevention and Population Sciences, Roswell Park Cancer Institute, Buffalo, NY, USA; ^21^ Department of Gynecology and Gynecologic Oncology, Kliniken Essen-Mitte, Essen, Germany; ^22^ Department of Gynecology and Gynecologic Oncology, Dr. Horst Schmidt Kliniken Wiesbaden, Wiesbaden, Germany; ^23^ Zentrum für Gynäkologische Onkologie, Kiel, Germany; ^24^ Department of Obstetrics and Gynaecology, Hannover Medical School, Hannover, Germany; ^25^ Women’s Cancer Program at the Samuel Oschin Comprehensive Cancer Institute, Cedars-Sinai Medical Center, Los Angeles, CA, USA; ^26^ Department of Health Sciences Research, Mayo Clinic, Rochester, MN, USA; ^27^ Department of Laboratory Medicine and Pathology, Mayo Clinic, Rochester, MN, USA; ^28^ Department of Gynecology, Rigshospitalet, University of Copenhagen, Copenhagen, Denmark; ^29^ Department of Virus, Lifestyle and Genes, Danish Cancer Society Research Center, Copenhagen, Denmark; ^30^ Department of Public Health Sciences, The University of Virginia, Charlottesville, VA, USA; ^31^ Department of Obstetrics and Gynecology, Duke University Medical Center, Durham, NC, USA; ^32^ Obstetrics and Gynecology Epidemiology Center, Brigham and Women’s Hospital and Harvard Medical School, Boston, MA, USA; ^33^ Department of Epidemiology, Harvard School of Public Health, Boston, MA, USA; ^34^ Department of Gynecology and Obstetrics, Haukeland University Hospital, Bergen, Norway; ^35^ Centre for Cancer Biomarkers, Department of Clinical Science, University of Bergen, Bergen, Norway; ^36^ Department of Population Health, QIMR Berghofer Medical Research Institute, Brisbane, QLD, Australia; ^37^ Gynaecological Oncology Department, Mercy Hospital for Women, Melbourne, VIC, Australia; ^38^ Department of Obstetrics and Gynecology, Oregon Health & Science University, Portland, OR, USA; ^39^ Knight Cancer Institute, Oregon Health & Science University, Portland, OR, USA; ^40^ Beatson West of Scotland Cancer Centre, Glasgow, UK; ^41^ Division of Gynecologic Oncology, Princess Margaret Hospital, University Health Network, Toronto, Ontario, Canada.; ^42^ British Columbia’s Ovarian Cancer Research (OVCARE) Program, Vancouver General Hospital, BC Cancer Agency and University of British Columbia, British Columbia, Canada; ^43^ Departments of Pathology and Laboratory Medicine, Obstetrics and Gynaecology and Molecular Oncology, The University of British Columbia, Vancouver, British Columbia, Canada; ^44^ British Columbia’s Ovarian Cancer Research (OVCARE) Program, Department of Molecular Oncology, British Columbia Cancer Research Centre, Vancouver, British Columbia, Canada; ^45^ Peter MacCallum Cancer Center, The University of Melbourne, Australia; ^46^ Department of Gynaecological Oncology, Westmead Hospital, Sydney, NSW, Australia; ^47^ Centre for Cancer Genetic Epidemiology, Department of Public Health and Primary Care, University of Cambridge, Strangeways Research Laboratory, Worts Causeway, Cambridge, UK

**Keywords:** ovarian cancer outcome, genetic association, gene regulation, meta-analysis

## Abstract

We previously identified associations with ovarian cancer outcome at five genetic loci. To identify putatively causal genetic variants and target genes, we prioritized two ovarian outcome loci (1q22 and 19p12) for further study. Bioinformatic and functional genetic analyses indicated that *MEF2D* and *ZNF100* are targets of candidate outcome variants at 1q22 and 19p12, respectively. At 19p12, the chromatin interaction of a putative regulatory element with the *ZNF100* promoter region correlated with candidate outcome variants. At 1q22, putative regulatory elements enhanced *MEF2D* promoter activity and haplotypes containing candidate outcome variants modulated these effects. In a public dataset, *MEF2D* and *ZNF100* expression were both associated with ovarian cancer progression-free or overall survival time. In an extended set of 6,162 epithelial ovarian cancer patients, we found that functional candidates at the 1q22 and 19p12 loci, as well as other regional variants, were nominally associated with patient outcome; however, no associations reached our threshold for statistical significance (*p*<1×10^-5^). Larger patient numbers will be needed to convincingly identify any true associations at these loci.

## INTRODUCTION

Most women diagnosed with stage III-IV ovarian cancer will die from the disease [1]. Although there has been improvement in ovarian cancer survival time, developments in chemotherapy have not contributed to a substantial decrease in deaths from ovarian cancer [2]. Genetic markers that identify women with poor prognosis or who are more likely to respond to specific treatment regimens could help improve outcomes by individualization of therapy. To this end, we recently performed a genome-wide association analysis of ovarian cancer patients treated with chemotherapy. We analyzed European ancestry patients based on their treatment regime and using a threshold of *p* ≤ 1×10^-5^ we found five variants that associated with either progression free survival (PFS) or overall survival (OS): rs4910232 (11p15.3) and rs3795247 (19p12), associated with PFS in patients who had cytoreductive surgery for first-line treatment regardless of chemotherapy type (referred to as the “any chemotherapy” group, *n* = 4,095); rs6674079 (1q22) associated with OS in the any chemotherapy group (*n* = 4,426); and rs7950311 (11p15.4) and rs2549714 (16q23) associated with OS in patients known to have received first-line treatment of cytoreductive surgery and ≥4 cycles of paclitaxel and carboplatin intravenously at 3-weekly intervals (referred to as the “standard chemotherapy” group, *n* = 1,799) [3].

All five variants associated with ovarian cancer patient outcome, and other candidate variants correlated by linkage disequilibrium (*r*^2^ > 0.4), are located in non-coding regions of the genome. The potential regulatory targets of these variants are not obvious, especially as functional elements can target genes over 1 Mb away through long-range chromatin interactions [4, 5]. To overcome this barrier and identify target genes, we used available bioinformatic data to prioritize two ovarian cancer outcome loci (1q22 and 19p12) for analysis and performed chromatin conformation capture and other functional genetic approaches to identify variants that regulate gene expression. We also performed further testing of genetic associations using additional Ovarian Cancer Association Consortium (OCAC) patients in a new meta-analysis of patient outcome.

## RESULTS

### *ZNF100* is a potential regulatory target of candidate outcome variants at the 19p12 locus

The five variants associated with ovarian cancer patient outcome and additional candidate outcome variants (correlated by linkage disequilibrium, *r*^2^ > 0.4) were assessed for effects on gene expression using publicly available data. In The Cancer Genome Atlas (TCGA) study of ovarian tumor samples (*n* = 386), no candidate outcome variants significantly associated with expression of genes located within 1 Mb of the lead variant (Bonferroni threshold *p* < 0.00016), although significant associations were observed between gene expression and variants that were not correlated with the lead outcome variants (Supplementary File 1). Analysis of normal ovarian samples (*n* = 85) from the Genotype-Tissue Expression (GTEx) Project [6], indicated that minor alleles of 13 candidate variants at the 19p12 locus associated with increased *ZNF100* expression after false discovery rate correction (q < 0.05, *p* < 1×10^-5^; Supplementary Table 1). No candidate variants at the other outcome loci significantly associated with gene expression (q < 0.05).

**Figure 1:**
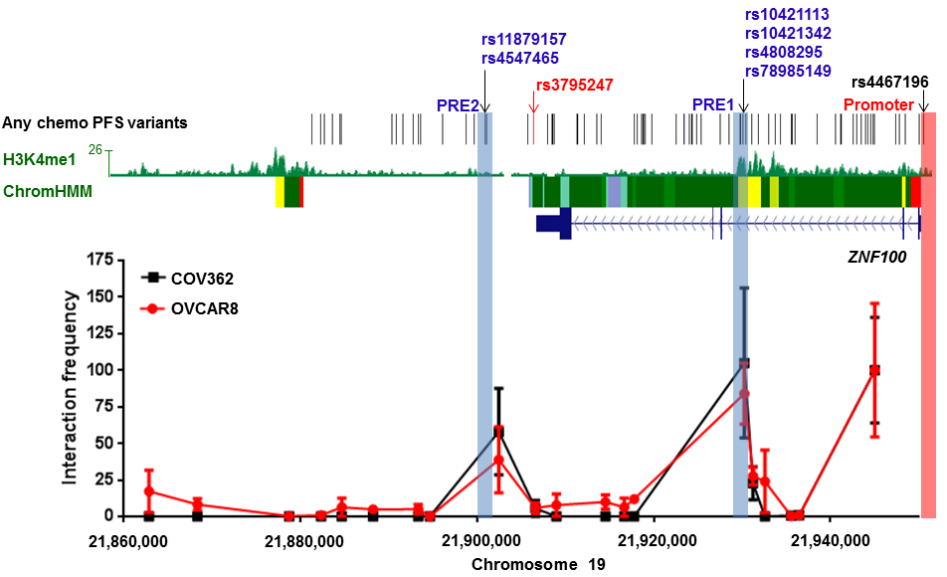
Two regions containing candidate all chemo PFS candidate variants interact with the *ZNF100* promoter in ovarian cancer cell lines at the 19p12 outcome locus The figure shows 3C analyses of interactions between HindIII fragments and the *ZNF100* promoter region (highlighted in pink) in COV362 and OVCAR8 cells. For each cell line, interaction frequencies were normalised to those of the fragment proximal to the promoter. Interaction frequencies from three independent biological replicates are shown (error bars represent standard error of the mean). The original variant associated with any chemotherapy PFS, rs3795247, is shown with correlated candidate outcome variants (*r*^2^ > 0.4) in black. Roadmap Consortium chromatin state segmentation for normal ovarian tissue using a Hidden Markov Model (Chrom HMM) is shown (red = active transcription start sites, dark green = weak transcription, green = strong transcription, green/yellow = genic enhancers, yellow = enhancers, aquamarine = ZNF gene repeats and turquoise = heterochromatin). H3K4Me1 modification in normal ovarian tissue is also indicated. Putative Regulatory Elements (PREs), and their coincident candidate variants, cloned into reporter gene constructs are highlighted in blue.

To determine if candidate variants at 19p12 interact with the *ZNF100* promoter region through long-range chromatin interactions, we performed chromatin conformation capture (3C) analyses in COV362 and OVCAR8 ovarian cancer cell lines. These cell lines were prioritized for study based on *ZNF100* expression in serous ovarian cancer cell lines (Supplementary Figure 1). Through 3C analysis across this outcome-associated locus, we identified two peaks of interaction, both containing candidate outcome variants (Figure 1).

### Putative regulatory elements (PREs) modulate *ZNF100* promoter activity

**Figure 2:**
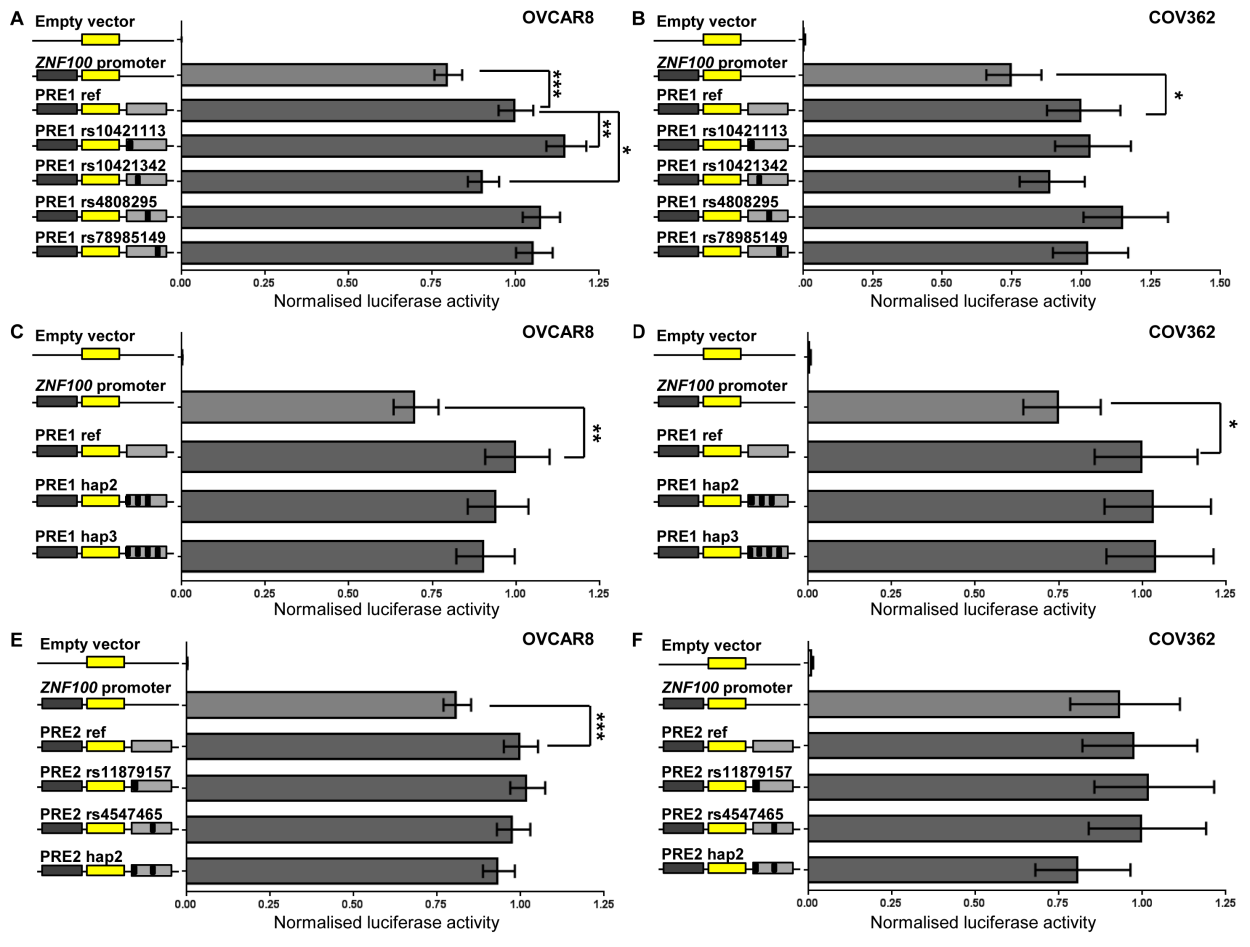
*ZNF100* PRE1 and PRE2 enhance promoter activity in OVCAR8 cells *ZNF100* PRE1 and PRE2 regions containing the major alleles of candidate outcome variants were cloned downstream of a *ZNF100*-promoter-driven luciferase construct (*ZNF100* promoter in figure) for the creation of reference (PRE1 ref and PRE2 ref) constructs. Minor allelic variants of the outcome variants were engineered into constructs and are designated by the rs ID of the corresponding variant and a black box in the construct schematic. Constructs containing haplotypic variants occurring in Europeans were also generated: PRE1 hap2 (minor allelic variants of all candidate variants except for rs4808295; 10% frequency in Europeans) and PRE1 hap3 (all minor allelic variants; 4% frequency in Europeans); and PRE2 hap2 (all minor allelic variants). Cells were transiently transfected with each of these constructs and assayed for luciferase activity after 24 h. Back-transformed data are shown and error bars denote 95% confidence intervals of experiments performed in triplicate for OVCAR8 cells and in quadruplicate for COV362 cells. P-values were determined by two-way ANOVA followed by Dunnett’s multiple comparisons test (*p < 0.05, **p < 0.01 and ***p < 0.001).

The two chromatin interacting regions have the potential to regulate *ZNF100* promoter activity. Functionality at the region most frequently interacting with the *ZNF100* promoter was further indicated by the presence, in normal ovary, of H3K4me1 histone modification (associated with enhancers) and an enhancer predicted by chromatin state segmentation (Figure 1). We defined the part of the interacting region marked by H3K4me1 and the predicted enhancer as *ZNF100* putative regulatory element (PRE1) (Figure 1). We cloned *ZNF100* PRE1, containing four candidate variants into a reporter gene construct containing the *ZNF100* promoter. A variant *ZNF100* promoter construct containing the minor allele of the rs4467196 candidate variant was also generated but this variant did not significantly affect promoter activity (*p* > 0.05, Supplementary Figure 2) and, thus, we used the promoter construct carrying the common allele for the luciferase analysis of PREs. The reference *ZNF100* PRE1 construct (containing the major alleles of the candidate variants) enhanced activity 1.25-1.43 fold over the construct containing the *ZNF100* promoter alone in OVCAR8 and COV362 cells (Figure 2A-2D). Two candidate variants in *ZNF100* PRE1 affected *ZNF100* promoter activity in OVCAR8 cells: the minor allele of rs10421113 increased promoter activity by 1.15 fold (*p* = 0.007; Figure 2A); and the minor allele of rs10421342 reduced activity by 1.10 fold (*p* = 0.04; Figure 2A). However, candidate variants did not affect promoter activity in COV362 cells (Figure 2B), nor did haplotypes containing these alleles have significant effects in either cell line (Figure 2C-2D).

At the second promoter-interacting region (Figure 1), one of the candidate variants (rs4547465) associated with *ZNF100* expression in ovarian tissue (Supplementary Table 1) and, thus, we cloned a region (*ZNF100* PRE2; Figure 1) containing rs4547465 and a neighboring candidate variant (rs11879157) into the reporter gene construct containing the *ZNF100* promoter. The reference *ZNF100* PRE2 increased promoter activity 1.23 fold in OVCAR8 cells (Figure 2E), but had no significant effect in COV362 cells (Figure 2F). None of the candidate variants affected *ZNF100* PRE2 activity in either cell line (Figure 2E-2F).

**Figure 3:**
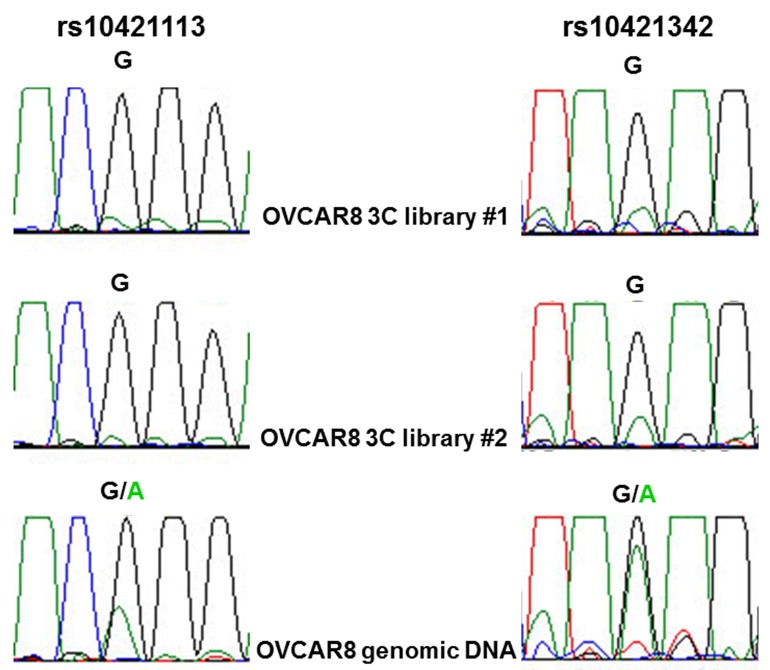
Allele-specific interaction between *ZNF100* PRE1 and *ZNF100* promoter region OVCAR8 cells are heterozygous for rs10421113 and rs10421342 candidate outcome variants. Allele-specific 3C analysis using PCR-based Sanger sequencing of two independent 3C libraries indicates that sequence containing the major G allele of both variants preferentially interacts with the *ZNF100* promoter.

The two potentially functional candidate variants in *ZNF100* PRE1, rs10421113 and rs10421342, were amenable to allele-specific 3C analysis in OVCAR8 cells due to their heterozygosity and proximity to a HindIII restriction site used in the 3C analysis. PCR-based Sanger sequencing indicated that the major (G) alleles of rs10421113 and rs10421342 are located in a region that preferentially interacts with the *ZNF100* promoter region in OVCAR8 cells (Figure 3). Notably, the minor (A) allele of rs10421113 weakens several binding motifs of CTCF (Supplementary Table 2), a transcription factor that is required for cell type-specific chromatin interactions [7]. Allele-specific 3C analysis of *ZNF100* PRE1 could not be performed in COV362 cells as this cell line was not heterozygous for rs10421113 and rs10421342. Allele-specific 3C analysis also could not be performed for *ZNF100* PRE2 as an amenable heterozygous candidate variant was not found in either OVCAR8 or COV362 cells.

### Candidate variants in an ovarian super-enhancer at 1q22 modify *MEF2D* promoter activity

**Figure 4:**
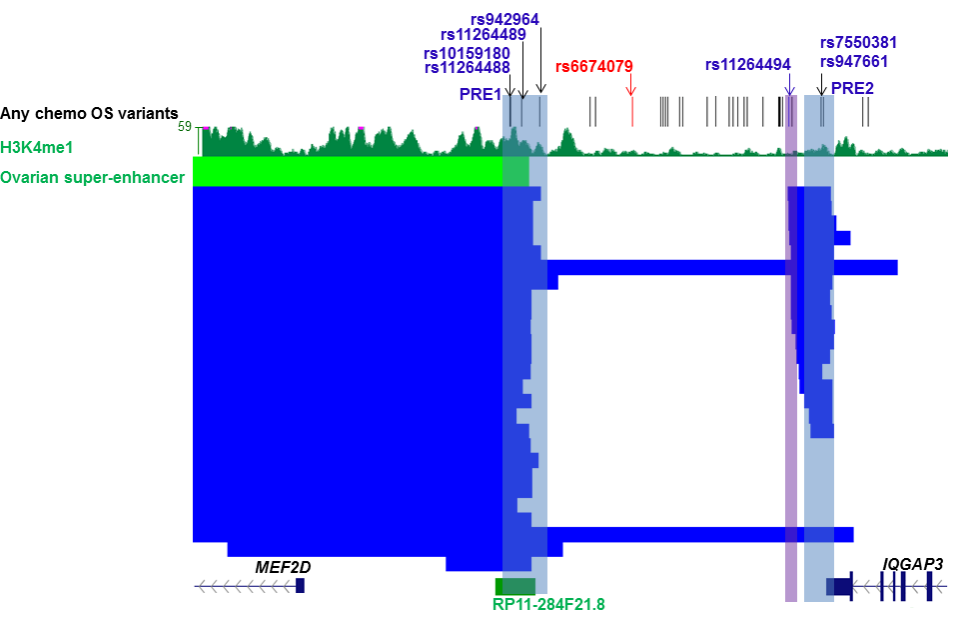
Candidate outcome variants at 1q22 are coincident with an ovarian super-enhancer that is predicted to target *MEF2D* The figure shows candidate outcome variants, H3K4Me1 modification in normal ovarian tissue and super-enhancers (ovarian super-enhancer is colored green and super-enhancers from other tissues blue) at the 1q22 ovarian outcome locus that are predicted to target MEF2D (Supplementary Table 3). Putative Regulatory Elements (PREs), and their coincident candidate variants, cloned into reporter gene constructs are highlighted in blue. The original lead variant associated with any chemotherapy OS, rs6674079, is shown with correlated candidate outcome variants (*r*^2^ > 0.4) in black and the lead variant from the new OncoArray analysis with all chemotherapy OS, rs11264494, in blue (region cloned into reporter gene constructs highlighted in purple).

Super-enhancers are dense clusters of enhancers and have been found to be enriched for disease associated variants in previous studies [8, 9]. We interrogated a catalogue of super-enhancers across 86 human cell and tissue types [9] and found candidate variants at the 1q22 outcome locus were coincident with super-enhancers present in multiple tissues (Supplementary Table 3), including normal ovary (Figure 4). A region containing four candidate outcome variants and substantial levels of H3K4Me1 in normal ovary was defined as *MEF2D* PRE1 (Figure 4). Three of these candidate outcome variants were also coincident with the ovarian super-enhancer which was predicted by the catalogue to regulate *MEF2D* through pairing of the super-enhancer with the nearest expressed gene in normal ovary [9]. Queries of GTEx eQTL data, indicated that the minor allele of one of *MEF2D* PRE1 candidate variants, rs11264489, was nominally associated (*p* = 0.032) with lower *MEF2D* expression in ovarian tissue (Supplementary Table 4). The three *MEF2D* PRE1 candidate variants in the ovarian super-enhancer were also located within RP11-284F21.8, a long non-coding RNA (lncRNA). Notably, lncRNAs are often associated with enhancer regions [10].

A multi-tissue super-enhancer region containing candidate outcome variants was observed near the 3’ end of *IQGAP3* at 1q22 (Figure 4). In particular, two candidate outcome variants adjacent to the 3’ end of *IQGAP3*, rs947661 and rs7550381, coincided with super-enhancers from 17 tissues, 16 of which were predicted to target *MEF2D* (Supplementary Table 3). This region was defined as PRE2. To further explore the potential target of these two variants, GTEx eQTL data for normal ovarian tissue were queried and nominally significant (*p*≤0.015; Supplementary Table 4) associations were observed between lower *MEF2D* expression and the minor alleles of both variants, but not with expression of the neighboring *IQGAP3* gene.

**Figure 5:**
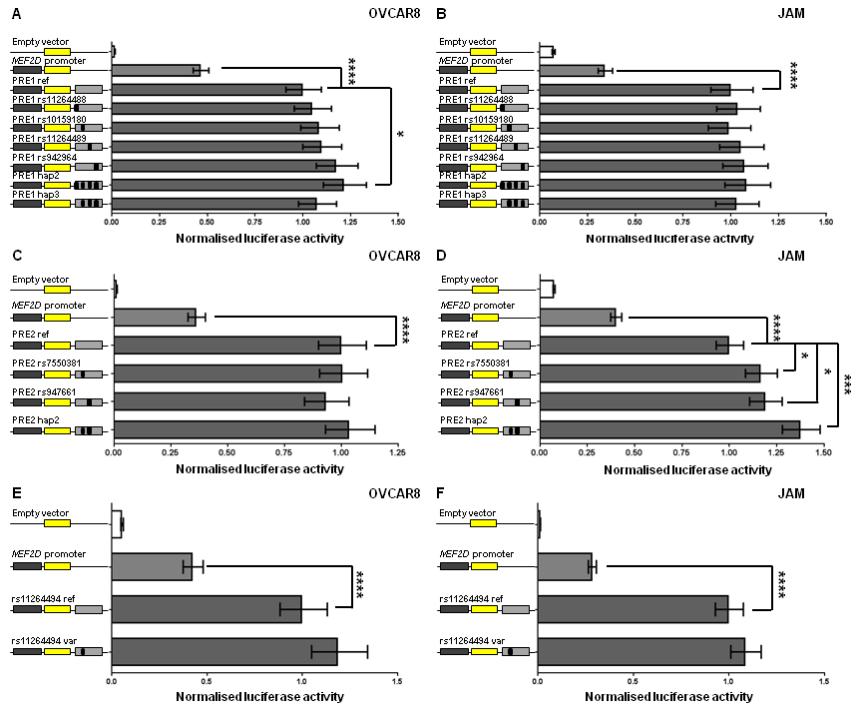
*MEF2D* regulatory regions act as enhancers and candidate outcome variants increase *MEF2D* PRE1 and PRE2 activity Putative *MEF2D* regulatory regions containing candidate outcome variants were cloned downstream of a *MEF2D*-promoter-driven luciferase construct (*MEF2D* promoter in figure). The reference PRE1, PRE2 and rs11264494 constructs contain the most common haplotypes or alleles in Europeans. Alternative alleles of candidate outcome variants were engineered into constructs and are designated by the rs ID of the corresponding variant and a black box in the construct schematic. Constructs containing other haplotypes were also generated: PRE1 hap2 (all alternative alleles of PRE1 ref; 35% frequency in Europeans) and hap3 (all alternative alleles of PRE1 ref except rs11264488; 15% frequency in Europeans); and PRE2 hap2 (all alternative alleles of PRE2 ref; 30% frequency in Europeans). Cells were transiently transfected with each of these constructs and assayed for luciferase activity after 24 h. Back-transformed data are shown and error bars denote 95% confidence intervals of replicate experiments performed in OVCAR8 and JAM cells. P-values were determined by two-way ANOVA followed by Dunnett’s multiple comparisons test (*p < 0.05, **p < 0.01, ***p < 0.001 and ****p < 0.0001).

The *MEF2D* promoter was too close to the candidate outcome variants for analysis of DNA chromatin interactions by 3C but the super-enhancer and GTEx eQTL data suggested that *MEF2D* is a regulatory target. To examine the effects of variants on activity of the *MEF2D* promoter, we tested the effects of the four candidate outcome variants in *MEF2D* PRE1 on promoter activity in OVCAR8 and JAM cells, the two ovarian cancer cell lines in which *MEF2D* was most highly expressed (Supplementary Figure 1). The reference *MEF2D* PRE1 construct, containing the most common European haplotype (1000 Genomes Project) of the four candidate variants, acted as an enhancer of promoter activity. Compared with a reporter gene construct containing the *MEF2D* promoter alone, the reference *MEF2D* PRE1 increased *MEF2D* promoter activity by 2.2-2.9 fold in OVCAR8 and JAM cells (Figure 5A-5B). In OVCAR8 cells, candidate variants did not individually show an effect but a haplotype (*MEF2D* PRE1 hap2) containing the alternate alleles of the variants in the reference construct had 1.21 fold (p = 0.03) greater activity than the reference *MEF2D* PRE1 (Figure 5A). It appears that the alternate (C) allele of rs11264488 is necessary for the haplotype effect as *MEF2D* PRE1 hap3, which does not contain this allele, has no effect on the PRE activity. Furthermore, this allele weakens the binding motif of FOXP1 (Supplementary Table 2), a transcriptional repressor [11] that appears to act as an oncogene in ovarian cancer [12]. In JAM cells, none of the candidate variants, or their haplotypes, affected *MEF2D* promoter activity (Figure 5B). *MEF2D* PRE2 also acted as an enhancer and increased *MEF2D* promoter activity 2.5-2.8 fold in OVCAR8 and JAM cells (Figure 5C-5D). The *MEF2D* PRE2 candidate variants, rs7550381 and rs947661, had no effect in OVCAR8 cells (Figure 5C), but in JAM cells their minor alleles were associated with 17% (*p* = 0.03) and 19% (*p* = 0.01), greater *MEF2D* PRE2 activity, respectively (Figure 5D). Notably, the minor allele (A) of rs7550381 strengthened two binding motifs of CTCF (Supplementary Table 2). The effect of these alleles appeared to be additive as the haplotype containing both (*MEF2D* PRE2 hap2) had 38% (*p* = 0.0002) greater activity (Figure 5D). We also cloned the promoter of the RP11-284F21.8 lncRNA into the *MEF2D* PRE1 and *MEF2D* PRE2 constructs to assess its effects but measurable levels of RP11-284F21.8 promoter activity were not detected (data not shown).

### *MEF2D* and *ZNF100* expression is associated with poorer ovarian cancer outcome

The Kaplan-Meier plotter (KM-plot) web-based application [13] was used to assess associations between *MEF2D* and *ZNF100* expression and outcome using data available from serous ovarian cancer patients. For *MEF2D*, no significant associations with OS were observed using data from three different probe sets (*n* = 453-1138; Supplementary Figure 3), but higher expression associated (*p*<0.05) with shorter PFS and the associations observed from the two probe sets with the largest number of samples (*n* = 1019) also passed a false discovery rate correction (q < 0.05) (Supplementary Figure 4). For *ZNF100*, higher expression was significantly associated with shorter OS (*p* = 0.013, *q* = 0.035; *n* = 453) but no association was observed with PFS (*n* = 397) (Supplementary Figure 5).

Analysis of 1q22 and 19p12 outcome loci using a denser genotyping platform and additional patients

To refine the genetic associations at the 1q22 and 19p12 outcome loci, we analyzed 1 Mb regions encompassing both lead variants using a denser genotyping platform (OncoArray [14]) than had been previously applied to the OCAC patient set [3]. Additionally, to perform analyses in the largest possible number of patients, we meta-analyzed data in an expanded patient set that included patients from our previous study [3]. After quality control and data cleaning, the following numbers of patients were included in meta-analysis: for OS, 6,160 with any chemotherapy (*N* = 4,426 previously [3]), 2,620 with standard chemotherapy (*N* = 1,799 previously [3]); for PFS, 5,596 with any chemotherapy (*N* = 4,095 previously [3]) and 2,352 with standard chemotherapy (*N* = 1,958 previously [3]). However, none of the associations tested reached our previous threshold for significance (*p* < 1.0×10^-5^ [3]) in any analysis (Table 1).

**Table 1:** Meta-analysis of 1q22 and 19p12 loci for genetic association with survival outcomes in ovarian cancer patients

Variant	Position (hg19)			OS any chemotherapy (n=6,160)		OS standard chemotherapy (n=2,620)		PFS any chemotherapy (n=5,596)		PFS standard chemotherapy (n=2,352)	
		Effect allele/Ref allele	EAF§	HR (95% CI)	*P*	HR (95% CI)	*P*	HR (95% CI)	*P*	HR (95% CI)	*P*
**1q22 region**											
rs6674079*	156486061	A/G	0.60	0.93 (0.88-0.98)	0.010	0.95 (0.88-1.04)	0.257	0.98 (0.93-1.03)	0.398	1.00 (0.93-1.08)	0.918
rs11264494^¶^	156493398	C/T	0.51	**0.91 (0.86-0.96)**	**2.6×10**^-4^	0.91 (0.84-0.99)	0.023	0.92 (0.88-0.97)	0.001	0.92 (0.86-0.99)	0.024
rs2274500^¶^	156850112	G/T	0.86	1.09 (0.98-1.21)	0.110	**1.24 (1.06-1.45)**	**0.007**	1.05 (0.95-1.16)	0.312	1.16 (1.00-1.35)	0.047
rs60989774^¶^	156513579	CT/C	0.38	1.08 (1.02-1.14)	0.006	1.08 (1.00-1.18)	0.061	**1.11 (1.05-1.17)**	**8.0×10**^-4^	1.11 (1.02-1.20)	0.011
rs59359496^¶^	156501450	T/G	0.08	1.04 (0.95-1.14)	0.422	1.07 (0.93-1.23)	0.340	1.13 (1.03-1.23)	0.007	**1.21 (1.07-1.38)**	**0.003**
rs947661^#^	156494916	A/G	0.36	1.10 (1.04-1.16)	8.2×10^-4^	1.09 (1.00-1.19)	0.050	1.04 (0.99-1.10)	0.118	1.04 (0.96-1.12)	0.367
rs10159180^#^	156480324	C/T	0.46	0.92 (0.87-0.97)	0.001	0.93 (0.86-1.01)	0.089	0.94 (0.89-0.98)	0.007	0.95 (0.88-1.02)	0.176
rs942964^#^	156481707	A/G	0.54	1.09 (1.03-1.15)	0.001	1.07 (0.99-1.16)	0.097	1.07 (1.02-1.12)	0.010	1.05 (0.98-1.13)	0.178
rs11264488^#^	156480288	C/G	0.56	1.08 (1.03-1.14)	0.004	1.05 (0.97-1.14)	0.194	1.07 (1.02-1.13)	0.005	1.05 (0.98-1.13)	0.171
rs11264489^#^	156480831	A/G	0.60	0.92 (0.87-0.97)	0.004	0.95 (0.87-1.03)	0.194	0.98 (0.93-1.03)	0.365	1.00 (0.93-1.08)	0.990
rs7550381^#^	156495001	A/G	0.60	0.92 (0.87-0.97)	0.004	0.94 (0.87-1.03)	0.181	0.96 (0.92-1.01)	0.156	0.98 (0.91-1.06)	0.604
**19p12 region**											
rs3795247*	21906428	T/C	0.90	0.90 (0.82-0.98)	0.021	0.80 (0.69-0.93)	0.003	0.89 (0.82-0.97)	0.007	0.83 (0.73-0.95)	0.009
rs79447188^¶^	22405414	T/C	0.03	**1.20-(1.05-1.37)**	**0.006**	1.26 (1.03-1.55)	0.024	1.13 (1.00-1.28)	0.044	1.10 (0.90-1.34)	0.342
rs4330044^¶^	21905838	T/C	0.10	1.12 (1.02-1.22)	0.021	**1.25 (1.08-1.44)**	**0.003**	1.13 (1.03-1.23)	0.007	1.20 (1.05-1.38)	0.009
rs35308521^¶^	21932095	G/GA	0.11	1.12 (1.02-1.23)	0.015	1.24 (1.08-1.43)	0.003	**1.14 (1.05-1.25)**	**0.002**	1.21 (1.06-1.38)	0.005
rs12463030^¶^	21947607	T/C	0.95	0.88 (0.76-1.01)	0.071	0.76 (0.60-0.95)	0.016	0.85 (0.74-0.97)	0.015	**0.72 (0.58-0.89)**	**0.002**
rs10421113^#^	21929885	T/C	0.83	1.02 (0.95-1.10)	0.575	1.11 (0.99-1.24)	0.065	1.04 (0.98-1.11)	0.227	1.11 (1.00-1.23)	0.047
rs10421342^#^	21930003	T/C	0.83	1.02 (0.95-1.10)	0.575	1.11 (0.99-1.24)	0.065	1.04 (0.98-1.11)	0.227	1.11 (1.00-1.23)	0.047

^§^Effect allele frequency based on samples from the OncoArray study.

*Lead outcome variant in our original study [3].

^¶^Lead outcome variant with a survival phenotype in the new meta-analysis.

^#^Functional candidate variant.

At 1q22, the previous lead variant, rs6674079, was only nominally significant for association with OS among patients with any chemotherapy: *p* = 0.01 (Table 1) *versus p* = 7.1×10^-6^ previously; the strongest nominal association at this locus for this group was with rs11264494 (*p* = 2.6×10^-4^, Table 1). Of the candidate variants in haplotypes with functional effects on *MEF2D* promoter activity, the strongest association was observed between PRE2 variant rs947661 and any chemotherapy OS (*p* = 8.2×10^-4^; Table 1). Other candidate variants from functional haplotypes also nominally associated with OS and PFS among patients treated with any chemotherapy, but no variant at 1q22 passed our significance threshold of *p* < 1×10^-5^ (Table 1). Linkage disequilibrium was observed between the previous lead candidate, rs6674079, and rs11264494 (*r*^2^ = 0.33 in Europeans) and also between rs11264494 and candidate variants from the haplotypes with functional effects on *MEF2D* promoter activity (*r*^2^ = 0.33-0.79).

At 19p12, the association of the previous lead variant, rs3795247, with any chemotherapy PFS also lost significance: *p* = 0.007 *versus p* = 1.05×10^-5^ previously (Table 1). The strongest nominal association at this locus was between rs35308521 and any chemotherapy PFS (*p* = 0.002, Table 1). For the two candidate variants that correlated with the allele-specific chromatin interaction with the *ZNF100* promoter region, nominal associations with outcome were observed only in the standard chemotherapy PFS analysis (Table 1). Linkage disequilibrium was observed between the previous lead candidate, rs3795247, and rs35308521 (*r*^2^ = 0.89) and between rs35308521 and the two variants that correlated with allele-specific looping with the *ZNF100* promoter region (*r*^2^ = 0.47).

### rs11264494 does not affect *MEF2D* promoter activity

Based on the results from the new genetic analysis, the variant with the strongest nominal association with outcome at either locus was rs11264494, located near the *MEF2D* PRE2 and proximal to several *MEF2D* super-enhancers (Figure 4). A region containing this variant was cloned in the luciferase reporter construct containing the *MEF2D* promoter. The region containing the (C) allele of rs11264494 enhanced *MEF2D* promoter activity 2.4-fold in OVCAR8 and 3.6-fold in JAM cells, but the T allelic variant of this SNP did not significantly affect this enhancer activity (Figure 5E-5F).

## DISCUSSION

We have performed follow-up analyses of the 1q22 and 19p12 ovarian cancer outcome loci to identify functional genetic variants and refine the genetic associations. At the 1q22 locus, candidate outcome variants were located in an ovarian super-enhancer and haplotypes containing these variants had differential effects on *MEF2D* promoter activity. At the 19p12 locus, an allele-specific chromatin interaction with the *ZNF100* promoter region was observed with two candidate outcome variants. Candidate functional variants at both loci nominally associated with survival outcome; however, neither these, nor other variants at either locus, passed our significance threshold (*p* < 1×10^-5^).

*MEF2D* encodes the myocyte enhancer factor 2D, a transcription factor that was initially found to be associated with muscle cell differentiation [15] but which also affects tumor development and progression [16]. Notably, in patients diagnosed with osteosarcoma, hepatocellular carcinoma or colorectal cancer, poorer prognosis is associated with greater *MEF2D* expression [16-18]. Using publicly available data, we also identified associations between greater *MEF2D* expression and shorter PFS in women with serous ovarian cancer. Consistent with these observations, the C allele of rs11264488, the functional variant most strongly associated with any chemotherapy PFS, appears to be a requisite component of the *MEF2D* PRE1 haplotype with the greatest enhancer effect on *MEF2D* promoter activity. However, as this variant was only nominally associated with any chemotherapy PFS, links between its molecular effect and a clinical phenotype remain speculative.

*ZNF100* encodes a zinc finger protein transcription factor of which little is known, although the literature indicates that this gene may have a role in cancer. *ZNF100* is among the 30 most hypermethylated genes in colorectal cancer [19] and is frequently mutated in multiple myeloma with mutated alleles preferentially expressed [20]. Knockdown screens have shown that *ZNF100* knockdown sensitizes gastrointestinal stromal tumor cells to sunitinib and imatinib treatment [21]. The 19p12-13 region is amplified in ovarian cancer and *ZNF100,* and other ZNF genes, show increased expression in tumors with these amplifications [22]. In a public dataset, we identified an association between greater *ZNF100* expression and shorter OS but we have not been able to find direct evidence that candidate outcome variants at 19p12 up-regulate *ZNF100* expression.

This study has several limitations which should be noted. The clinical analysis is based on observational data and a lack of treatment standardization in our any chemotherapy analyses may cloud genetic associations; however, we had hypothesized that the analysis of a more homogenous treatment group in our standard chemotherapy analyses could reveal variants with greater effects, thus increasing power to detect associations. Nevertheless, it has become apparent that very large numbers of samples are needed to detect patient outcomes that are associated with common genetic variation. In the context of this study, 11,001 survival events are needed to detect with 80% power at *p* < 1×10^-5^ a variant with a minor allele frequency of 0.4 that confers a HR of 1.1. Therefore, though we cannot rule out associations between genetic variation at 1q22 and 19p12 with ovarian cancer patient outcome, larger patient numbers will be needed to identify true associations.

## MATERIALS AND METHODS

### TCGA eQTL analyses in ovarian cancer samples

Data from ovarian cancer patients were accessed from The Cancer Genome Atlas (TCGA; https://tcga-data.nci.nih.gov/). Tumor gene expression microarray data (Agilent G4502A platform) and copy-number information were downloaded through the public access TCGA portal. Germline genotypes (Affymetrix 6.0 arrays) were downloaded through the TCGA controlled access portal and QC performed. Variants were excluded for call rate < 95%, MAF < 1% or deviations from HWE significant at 10^-4^. Thirteen samples were excluded for low overall call rate ( < 95%), heterozygosity > 3 standard deviations from the mean or non-female sex status (X-chromosome homozygosity > 0.2). A pair of samples were identified as duplicates by identity-by-state probabilities = 0.99) and the sample with the lower call rate was excluded. Overall there were 386 TCGA patients with complete genotype, gene expression and copy-number data included in the analysis. Gene expression for genes 500kb upstream and downstream of each locus were adjusted for somatic copy number variation, as previously described [23]. The associations between genotype and adjusted expression for each gene were evaluated using linear regression models by PLINK [24].

### Cell lines

ES-2, COV318, COV362 cells were cultured in Dulbecco’s Modified Eagle Medium and non-essential amino acids; FUOV1 cells were cultured in DMEM/F12 with L-Glutamine; and JAM and OVCAR8 cells were cultured in RPMI1640 with L-glutamine. All cell lines were cultured with 10% fetal calf serum and penicillin/streptomycin at 37°C in a humidified 5% CO_2_ atmosphere. Cell lines were stored in liquid nitrogen vapor phase with *Mycoplasma* testing and short tandem repeat profiling performed for cell line authentication prior to storage.

### Real-time PCR (qPCR)

RNA was extracted from cells using Trizol (Life Technologies) and cDNA synthesised using the SuperScript III First-Strand Synthesis System (Life Technologies) according to the manufacturer’s instructions. mRNA expression was quantified by real-time PCR reactions using Syto9 and qPCR primers (Supplementary Table 5) with normalisation by *ACTB* expression.

### Chromatin conformation capture (3C)

3C libraries were generated from OVCAR8 and COV362 cells using HindIII (New England Biolabs) as described previously [25]. 3C interactions were quantified by qPCR with primers designed within the HindIII restriction fragments spanning the 19p12 outcome locus (Supplementary Table 5). qPCR was performed as described previously [26] using three independent 3C libraries from each cell line with each experiment quantified in duplicate. Two BAC clones (RP11-120C22 and RP11-420K14: BACPAC Resource Center) covering the 19p12 outcome locus were used to create an artificial library of ligation products in order to normalize for PCR efficiency. As an internal control, interaction frequencies were normalized to that of the HindIII fragment immediately upstream of the promoter/bait fragment. To perform allele-specific 3C, DNA was amplified by PCR of two independent 3C libraries using the bait and a reverse primer specific to a PRE1 fragment containing rs10421113 and rs10421342 (Supplementary Table 5). Sanger sequencing (Australian Genome Research Facility, Brisbane) was performed to determine the alleles present in the interacting PRE1 fragment.

### Reporter gene vector construction

*MEF2D* and *ZNF100* promoter-driven luciferase reporter constructs were generated by inserting 2119 and 703 bp of DNA PCR-amplified from the respective promoters into the KpnI and HindIII sites of pGL3-Basic. To assist cloning of PREs downstream of the *Firefly* luciferase gene in pGL3-Basic, additional restriction sites had been introduced in a modified pGL3-Basic construct [27] and AgeI and SalI were then used to insert PREs into pGL3-Basic. For *ZNF100* PRE1 and PRE2, a 1010 bp fragment was PCR generated using primers engineered with AgeI and SbfI sites and a 463 bp fragment (hg19, chr19:21900737-21901199) synthesized with terminal AgeI and SbfI sites (Integrated DNA Technologies, Singapore) for cloning into a modified pGL3-*ZNF100* promoter construct, respectively. For *MEF2D* PRE1 and PRE2, a 1917 bp fragment and a 1723 bp fragment were also PCR generated as above and cloned into a modified pGL3-*MEF2D* promoter construct, respectively. The 477 bp rs11264494 construct (hg19, chr1:154693242-156493718) was also synthesized by Integrated DNA Technologies and cloned as above. The minor alleles of individual variants were introduced into cloned sequences, containing the major alleles of any other causal candidate variants, by overlap extension PCR. Common haplotypes predicted to be present in Europeans, from analyses of 1,000 Genomes Project data accessed *via* the LDlink web-based application [28], were also generated. All constructs were sequenced to confirm variant incorporation (AGRF, Brisbane, Australia). PCR cloning and sequencing primers are listed in Supplementary Table 5.

### Reporter assays

COV362 and OVCAR8 cells were transfected with equimolar amounts of luciferase reporter plasmids and 50 ng of pRLTK transfection control plasmid with Lipofectamine 2000 (Life Technologies). The total amount of transfected DNA was kept constant at 600 ng for each construct by adding pUC19 as a carrier plasmid. Luciferase activity was measured 24 h post-transfection by the Dual-Glo Luciferase Assay System (Promega). To correct for differences in transfection efficiency or cell lysate preparation, *Firefly* luciferase activity was normalized to *Renilla* luciferase. All promoter constructs had greater activity than the empty pGL3-Basic construct which was used as a negative control. Data were log-transformed and statistical significance was tested by two-way ANOVA followed by Dunnett’s multiple comparisons test in GraphPad Prism.

### Patient samples and genotyping

Genotype analyses were restricted to invasive epithelial ovarian cancer patients of European ancestry with detailed chemotherapy and clinical follow-up for disease progression and survival following first-line treatment from OCAC and TCGA (Supplementary Table 6). PFS was defined as the interval between the date of histological diagnosis and the first confirmed sign of disease progression or death; OS was the interval between the date of histological diagnosis and death from any cause. Criteria for progression for OCAC patients have been previously described [29]. TCGA genotype data were downloaded through the TCGA data portal and assessed for ancestral outliers to determine those of European descent. Genotype data for analysis of variants at the 19p12 and 1q22 loci were available from OCAC, with samples being genotyped with the Illumina Infinium OncoArray-500K beadchip. Duplicate pairs of samples and first degree relative pairs were identified and the sample with the lower call rate was removed as appropriate. Genotype, detailed clinical follow-up and pathology data were available for 5,508 EOC patients from 24 OCAC sites. Meta-analyzed data included OncoArray dataset described above, the iCOGS (*n* = 317) samples which were independent to the OncoArray genotyped patient set) and TCGA datasets (*n* = 337) [3, 30]. In each case imputation was carried out using SHAPEIT and IMPUTE2 [31] and the 1000 Genomes phase 3 reference dataset, NCBI build b37, (October 2014 release). All studies received approval from their respective human research ethics committees, and all OCAC participants provided written informed consent.

### Statistical analysis

The associations between genotypes and PFS and OS were assessed for patients in the standard chemotherapy subset and the less restrictive any chemotherapy group. Patients who had an interval of > 12 months between the date of histological diagnosis and DNA collection were excluded from the analysis to avoid survival bias. Cox regression models adjusted for residual disease (nil *vs*. any), tumor stage (FIGO stage I-IV), histology (serous, mucinous, endometrioid, clear cell, other epithelial), tumor grade (low *vs*. high), age at diagnosis (OS only), nine European ancestry principal components, and stratified by study, were used to obtain the per-allele hazard ratio HR and standard error for each variant. We tested the proportional hazards assumption for the adjusted variables and stratified by those that violated the assumption, as described previously [30]. The effect estimates in the largest available dataset using data from different genotyping platforms were derived from fixed effects meta-analysis of platform-specific estimates. We evaluated associations with OS and PFS for each variant (minor allele frequency > 0.02, imputation quality INFO score > 0.9) within a 1 Mb region encompassing the original lead variants at the 19p12 and 1q22 loci. All tests for association were two-tailed and performed using STATA SE v. 13 (Stata Corp., USA) and the R project for Statistical Computing version 3.2.2 (http://www.r-project.org/).

## SUPPLEMENTARY MATERIALS FIGURES AND TABLES






