# Supplementary material for: Analyses of germline variants associated with ovarian cancer survival identify functional candidates at the 1q22 and 19p12 outcome loci
Source: Oncotarget. 2017 Jun 15;8(39):64670–84. doi: 10.18632/oncotarget.18501 (PMC5630285; doi:10.18632/oncotarget.18501)
Supplement: Supplementary file 1 [file oncotarget-08-64670-s001.pdf]

Analyses of germline variants associated with ovarian cancer survival identify functional candidates at the 1q22 and 19p12 outcome loci

Supplementary Material

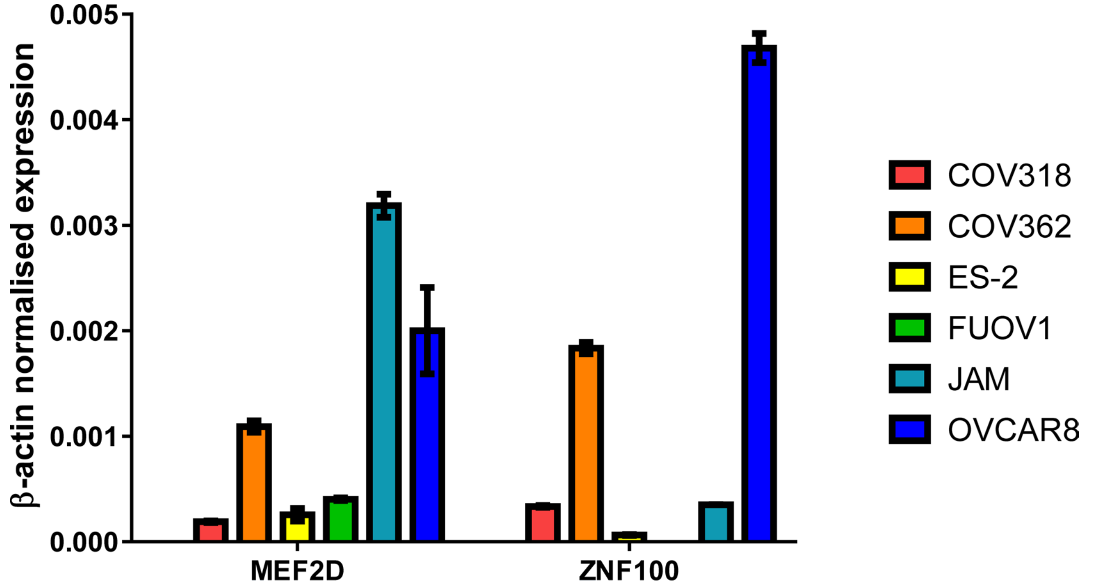

Supplementary Figure 1: Real-time PCR analysis of MEF2D and ZNF100 expression in six serous ovarian cancer cell lines. cDNA from six serous ovarian cancer cell lines was used in real-time PCR expression assays of *MEF2D* and *ZNF100* expression. *ACTB* (β-actin) expression was also measured and used as an internal control for normalisation. Real-time PCR assays were performed with duplicates and error bars indicate standard deviation.

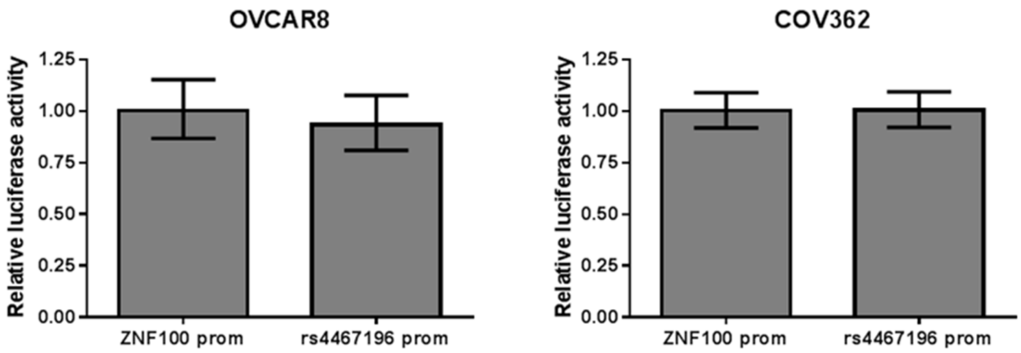

Supplementary Figure 2: A candidate outcome variant in the ZNF100 promoter (rs4467196) does not affect reporter gene activity in OVCAR8 or COV362 cells. A *ZNF100* promoter region containing the major allele of the rs4467196 candidate outcome SNP was cloned upstream of the *Firefly* luciferase gene in pGL3-Basic (ZNF100 prom). A construct containing the minor allele of rs4467196 was also generated (rs4467196 prom). Cells were transiently transfected with each of these constructs and assayed for luciferase activity after 24 h. Luciferase assays were performed using data from three experiments and error bars indicate 95% confidence intervals. Luciferase activity was compared using two-way ANOVA but no significant differences ($p<0.05$) were observed.

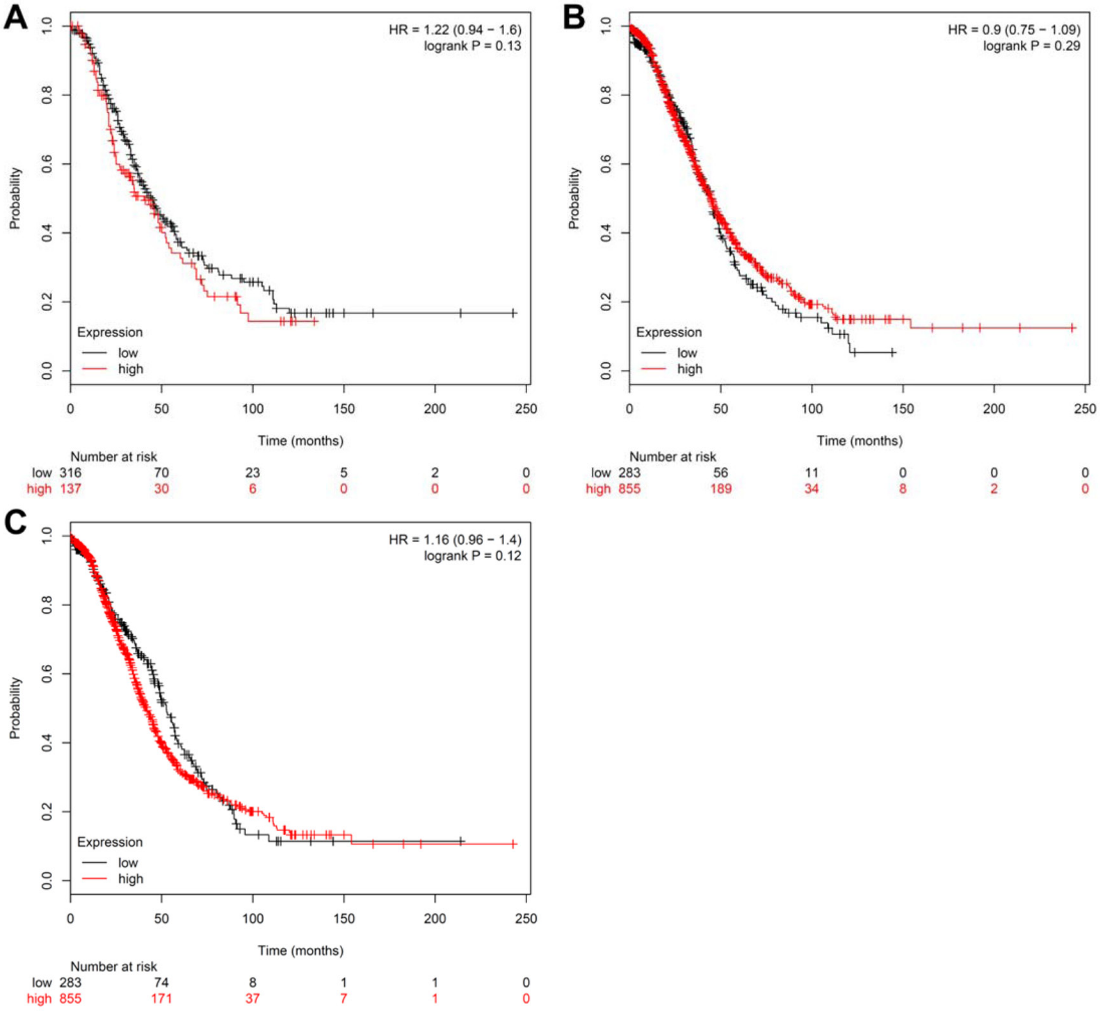

Supplementary Figure 3: MEF2D expression is not associated with OS in women with ovarian cancer. *MEF2D* expression data from serous ovarian cancer tumors was available for three Affymetrix probe sets: A) 225641 at (n=453); B) 203003 at (n=1138); and C) 203004 s at (n=1138). Patients were divided into high and low expression groups for each percentile between the lower and upper quartiles and associations with survival were performed using a Cox regression analysis. The best performing threshold was used as the final cut-off in the Kaplan-Meier survival plot and significance is indicated by a log-rank p-value.

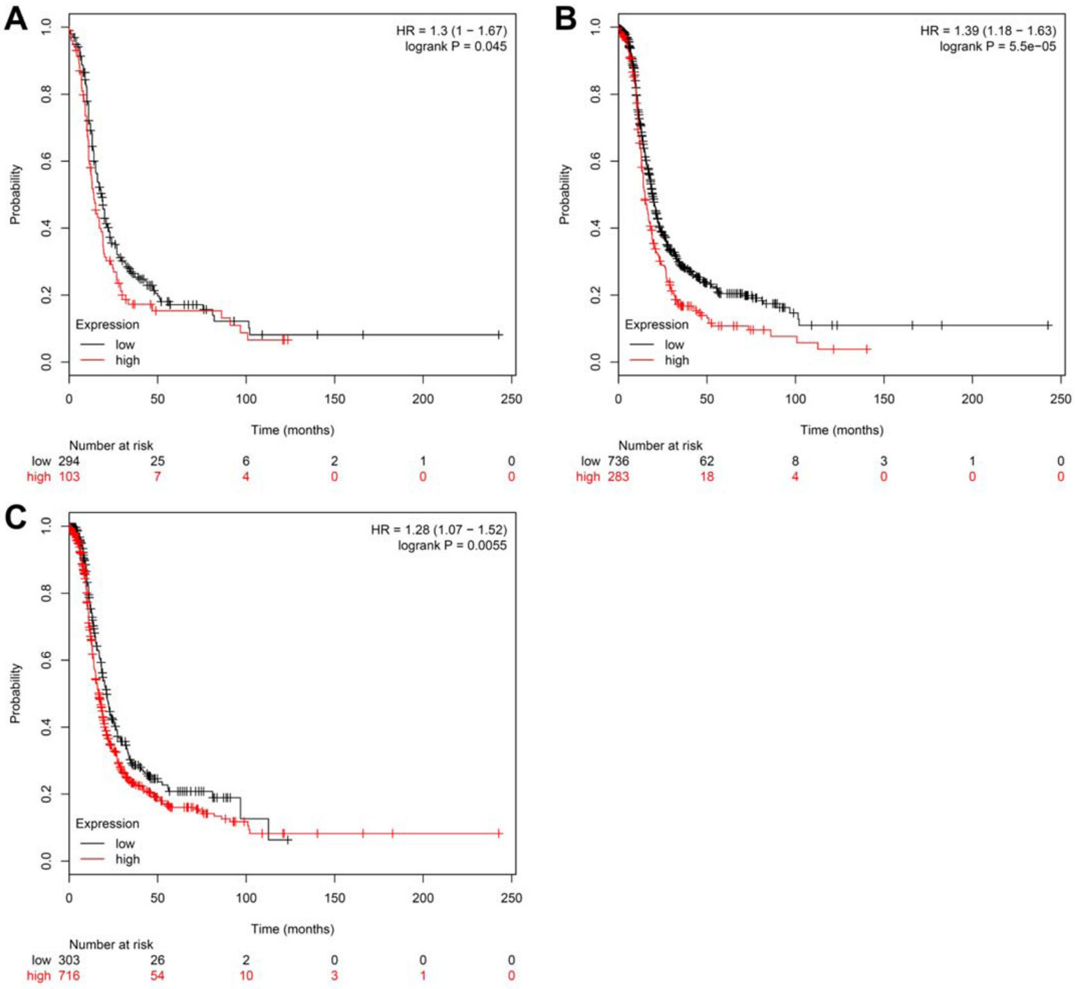

Supplementary Figure 4: MEF2D expression is associated with PFS in women with ovarian cancer. *MEF2D* expression data from serous ovarian cancer tumors was available for three Affymetrix probe sets: A) 225641 at (n=397); B) 203003 at (n=1019); and C) 203004 s at (n=1019). Patients were divided into high and low expression groups for each percentile between the lower and upper quartiles and associations with survival were performed using a Cox regression analysis. The best performing threshold was used as the final cut-off in the Kaplan-Meier survival plot and significance is indicated by a log-rank p-value.

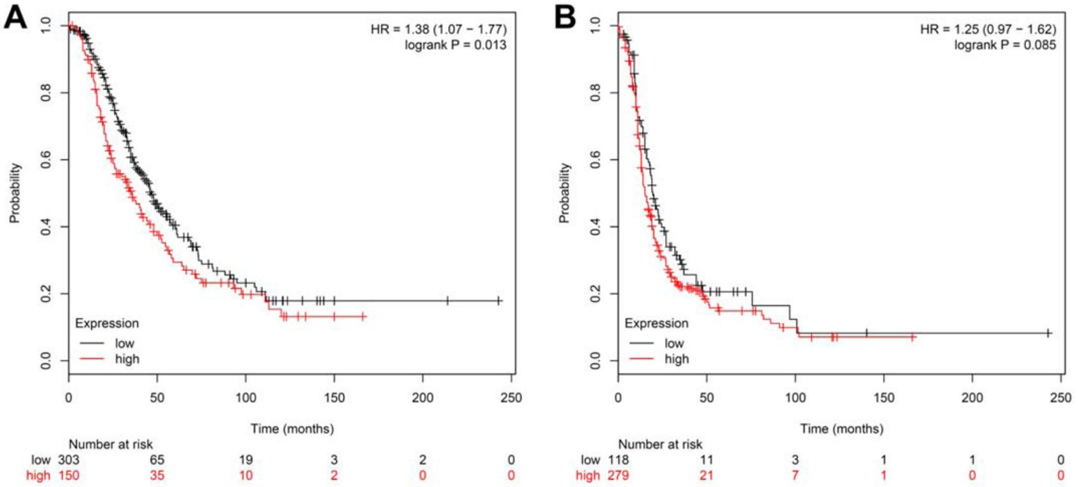

Supplementary Figure 5: ZNF100 expression is associated with OS in women with ovarian cancer. *ZNF100* expression data from serous ovarian cancer tumors was available for one Affymetrix probe set: 238791 at. Associations between expression and (A) OS (n=453) and (B) PFS (n=397) were tested. Patients were divided into high and low expression groups for each percentile between the lower and upper quartiles and associations with survival were performed using a Cox regression analysis. The best performing threshold was used as the final cut-off in the Kaplan-Meier survival plot and significance is indicated by a log-rank p-value.

For Supplementary Tables see in Supplementary Files.
