# Supplementary material for: Analyses of germline variants associated with ovarian cancer survival identify functional candidates at the 1q22 and 19p12 outcome loci
Source: Oncotarget. 2017 Jun 15;8(39):64670–84. doi: 10.18632/oncotarget.18501 (PMC5630285; doi:10.18632/oncotarget.18501)
Supplement: Supplementary file 2 [file oncotarget-08-64670-s002.docx]

**Supplementary Tables**

**Supplementary Table 1. Candidate outcome variants at the 19p12 locus significantly associated (q<0.05) with greater *ZNF100* expression in normal ovarian tissue (GTEx, n=85).**

| **SNP** | **PRE^*^ location** | **p-value** |
| --- | --- | --- |
| rs10469371 | - | 2.201E-06 |
| rs4547465 | 2 | 2.188E-06 |
| rs8111464 | - | 2.190E-06 |
| rs8105002 | - | 2.209E-06 |
| rs4613201 | - | 2.201E-06 |
| rs10421113 | 1 | 2.201E-06 |
| rs10421342 | 1 | 2.201E-06 |
| rs4808295 | 1 | 2.201E-06 |
| rs10423041 | - | 2.201E-06 |
| rs4809175 | - | 2.201E-06 |
| rs7258859 | - | 1.360E-06 |
| rs10412225 | - | 2.319E-06 |
| rs11085492 | - | 2.201E-06 |

^*^PRE: putative regulatory element

**Supplementary Table 2. Alteration of transcription factor binding position weight matrices (PWMs) by alleles of candidate variants.**

| **Candidate variant**  **(reference/alternative allele)** | **Location** | **PWM^*^** | **Reference allele score^*^** | **Alternative allele score^*^** |
| --- | --- | --- | --- | --- |
| rs4467196  (C/A) | *ZNF100* promoter | - | - | - |
| rs10421113 | *ZNF100* PRE1 | GATA_disc3 | 14.8 | 2.9 |
| (G/A) |  | NRSF_disc8 | 11 | 10.8 |
|  |  | PRDM1_known1 | -5 | 7 |
| rs10421342 | *ZNF100* PRE1 | CTCF_disc1 | 3.6 | 9.5 |
| (G/A) |  | CTCF_disc3 | 4.7 | 16.6 |
|  |  | CTCF_disc8 | -0.1 | 11.6 |
|  |  | CTCF_known1 | 2.5 | 9.9 |
|  |  | Roaz_1 | -9.1 | -20.6 |
|  |  | SMC3_disc1 | 2.5 | 10.3 |
|  |  | Zec | 5.5 | -6.1 |
| rs4808295 | *ZNF100* PRE1 | Egr-1_known4 | 1.3 | 13.3 |
| (A/G) |  | Egr-1_known5 | 3.7 | 10.9 |
|  |  | FXR_2 | 11.4 | 9.1 |
|  |  | GLI | 1 | 11.4 |
|  |  | Hbp1 | 12 | 4 |
|  |  | Hoxb3 | 9.9 | 11.9 |
|  |  | Nr2f2 | 8.9 | 11.2 |
|  |  | Pax-8_1 | 10.1 | 8.9 |
| rs78985149 | *ZNF100* PRE1 | Myc_known1 | 4.2 | 12.2 |
| (T/C) |  | Myc_known7 | 1.6 | 13.5 |
| rs11879157 | *ZNF100* PRE2 | GR_disc4 | 1 | 12.9 |
| (C/T) |  | TATA_disc1 | -0.5 | 11.5 |
|  |  | THAP1_disc2 | -5.4 | 6.5 |
|  |  | YY1_known2 | 6.4 | 11.7 |
|  |  | YY1_known3 | 7.9 | 12.3 |
|  |  | YY1_known4 | 4.9 | 16.8 |
| rs4547465 | *ZNF100* PRE2 | Arid5b | 11.9 | 13.1 |
| (G/A) |  | CDP_1 | -0.7 | 11.2 |
|  |  | Foxp1 | -10.2 | -9.2 |
|  |  | HDAC2_disc3 | 11.3 | 9.5 |
| rs942964  (G/A) | *MEF2D* PRE1 | GR_known3 | 1.4 | 11 |
| rs11264489 | *MEF2D* PRE1 | Hic1_2 | 8.5 | 11.6 |
| (A/G) |  | Mxi1_disc1 | 12.7 | 13.6 |
|  |  | RFX5_disc1 | 9.6 | 12.1 |
|  |  | SREBP_disc1 | 11.1 | 13.2 |
|  |  | STAT_known4 | 8.2 | 14.4 |
|  |  | STAT_known6 | -8.7 | 3.3 |
|  |  | STAT_known7 | 8.2 | 11.9 |
| rs10159180  (T/C) | *MEF2D* PRE1 | E2F_disc6 | 12.7 | 9.7 |
| rs11264488 | *MEF2D* PRE1 | Cdx2_2 | 10.5 | 11.6 |
| (C/G) |  | Foxp1 | 5.1 | 10.5 |
|  |  | HDAC2_disc6 | 17 | 10.6 |
|  |  | HNF1_7 | 9.3 | 12.3 |
|  |  | Sox_19 | 10.2 | 11.3 |
|  |  | Sox_2 | 16.1 | 16.5 |
|  |  | p300_disc5 | 14.1 | 14.3 |
| rs7550381 | *MEF2D* PRE2 | AP-2_disc2 | 12 | 13 |
| (G/A) |  | AP-2_known4 | 14 | 14.6 |
|  |  | BDP1_disc1 | 3.6 | 3 |
|  |  | CTCF_disc7 | 10.6 | 13.8 |
|  |  | CTCF_known1 | 4.8 | 9.5 |
|  |  | EBF_disc1 | 10.7 | 13.4 |
|  |  | EBF_disc2 | 12.1 | 12.6 |
|  |  | EBF_known1 | 10.8 | 13.5 |
|  |  | EBF_known2 | 4 | 15.2 |
|  |  | GR_disc6 | 12.7 | 1.9 |
|  |  | PU.1_disc3 | 12.4 | 12.5 |
|  |  | PU.1_known2 | -13 | -2.5 |
|  |  | VDR_2 | 13 | 9.5 |
|  |  | Zic_4 | 13 | 11 |
| rs947661 | *MEF2D* PRE2 | Evi-1_2 | -5 | -1.6 |
| (G/A) |  | Irf_known6 | 0.8 | 4.4 |
|  |  | KAP1_disc2 | -20.2 | -8.4 |
|  |  | Nkx2_11 | 12.5 | 7.9 |
|  |  | Pax-4_4 | 11 | 12 |
| rs11264494 | - | Crx_1 | 10.9 | 11.3 |
| (C/T) |  | Pitx2 | 16.2 | 15.6 |

^*^Accessed from Haploreg v4.1 (http://archive.broadinstitute.org/mammals/haploreg)

**Supplementary Table 3. Super-enhancers predicted to target *MEF2D* and coincident with candidate variants at 1q22.**

**Chr Start^*^ End^*^ PRE Tissue**

chr1 156425476 156481717 1 BI Hippocampus Middle

chr1 156425489 156481358 1 BI Brain Hippocampus Middle 150

chr1 156425489 156481358 1 BI Brain Hippocampus Middle 302

chr1 156425572 156481357 1 BI Brain Cingulate Gyrus

chr1 156425594 156481720 1 BI Brain Inferior Temporal Lobe

chr1 156425720 156498527 1 CD14

chr1 156425814 156482547 1 UCSD Psoas Muscle

chr1 156425839 156481284 1 UCSD Sigmoid Colon

chr1 156425855 156481316 1 UCSD Left Ventricle

chr1 156425857 156481259 1 BI Brain Angular Gyrus

chr1 156425860 156481289 1 UCSD Right Atrium

chr1 156425916 156481291 1 UCSD Lung

chr1 156425930 156481272 1 UCSD Esophagus

chr1 156425939 156480889 1 UCSD Gastric

chr1 156425948 156481289 1 UCSD Aorta

chr1 156426421 156481207 1 UCSD Ovary

chr1 156441114 156481261 1 UCSD Small Intestine

chr1 156444182 156481638 1 HSMMtube

chr1 156444667 156481327 1 NHLF

chr1 156444845 156481322 1 Fetal muscle

chr1 156449690 156481292 1 UCSD Spleen

chr1 156458712 156496436 1 Colon Crypt 1

chr1 156467034 156482747 1 HeLa

chr1 156477294 156481293 1 BI Brain Anterior Caudate

chr1 156493382 156495361 2 BI CD8 Memory 7pool

chr1 156493408 156495402 2 BI CD4 Memory Primary 7pool

chr1 156493423 156495629 2 Fetal Thymus

chr1 156493457 156496301 2 MM1S

chr1 156493513 156495490 2 GM12878

chr1 156493519 156495441 2 CD8 primary

chr1 156493523 156495333 2 Jurkat

chr1 156493524 156495393 2 BI CD34 Primary RO01536

chr1 156493536 156495527 2 CD56

chr1 156493728 156495520 2 RPMI-8402

chr1 156493800 156495310 2 UCSD Thymus

chr1 156493904 156494955 2 K562

chr1 156493935 156495434 2 UCSD Small Intestine

chr1 156494146 156495432 2 UCSD Sigmoid Colon

chr1 156494364 156495415 2 UCSD Spleen

chr1 156494455 156495519 2 BI CD34 Primary RO01549

^*^GRCh37/hg19

**Supplementary Table 4. Associations of candidate outcome variants at the 1q22 locus with *MEF2D* and *IQGAP3* expression in normal ovarian tissue (GTEx, n=85).**

| **SNP** | **PRE location** | **Gene** | **p-value** | **Gene** | **p-value** |
| --- | --- | --- | --- | --- | --- |
| rs11264488 | 1 | *MEF2D* | 0.180 | *IQGAP3* | 0.78 |
| rs10159180 | 1 | *MEF2D* | 0.180 | *IQGAP3* | 0.98 |
| rs11264489 | 1 | *MEF2D* | **0.032** | *IQGAP3* | 0.57 |
| rs942964 | 1 | *MEF2D* | 0.078 | *IQGAP3* | 0.93 |
| rs35425686 | - | *MEF2D* | N/A | *IQGAP3* | N/A |
| rs6427312 | - | *MEF2D* | 0.064 | *IQGAP3* | 0.91 |
| rs6674079 | - | *MEF2D* | **0.028** | *IQGAP3* | 0.56 |
| rs61813464 | - | *MEF2D* | 0.084 | *IQGAP3* | 0.78 |
| rs61813465 | - | *MEF2D* | 0.084 | *IQGAP3* | 0.78 |
| rs12084217 | - | *MEF2D* | 0.084 | *IQGAP3* | 0.78 |
| rs1750306 | - | *MEF2D* | 0.028 | *IQGAP3* | 0.56 |
| rs1778832 | - | *MEF2D* | **0.028** | *IQGAP3* | 0.56 |
| rs1750307 | - | *MEF2D* | **0.028** | *IQGAP3* | 0.56 |
| rs1750308 | - | *MEF2D* | **0.025** | *IQGAP3* | 0.60 |
| rs1778830 | - | *MEF2D* | **0.026** | *IQGAP3* | 0.57 |
| rs60711781 | - | *MEF2D* | N/A^*^ | *IQGAP3* | N/A |
| rs200223591 | - | *MEF2D* | N/A | *IQGAP3* | N/A |
| rs35361354 | - | *MEF2D* | N/A | *IQGAP3* | N/A |
| rs10908506 | - | *MEF2D* | 0.080 | *IQGAP3* | 0.80 |
| rs10908507 | - | *MEF2D* | 0.080 | *IQGAP3* | 0.81 |
| rs11264491 | - | *MEF2D* | 0.079 | *IQGAP3* | 0.88 |
| rs11264492 | - | *MEF2D* | 0.090 | *IQGAP3* | 0.81 |
| rs7533916 | - | *MEF2D* | **0.012** | *IQGAP3* | 0.74 |
| rs60322795 | - | *MEF2D* | **0.012** | *IQGAP3* | 0.74 |
| rs11264493 | - | *MEF2D* | **0.018** | *IQGAP3* | 0.67 |
| rs7544205 | - | *MEF2D* | **0.012** | *IQGAP3* | 0.74 |
| rs4661177 | - | *MEF2D* | **0.013** | *IQGAP3* | 0.75 |
| rs947661 | 2 | *MEF2D* | **0.018** | *IQGAP3* | 0.67 |
| rs7550381 | 2 | *MEF2D* | **0.014** | *IQGAP3* | 0.76 |
| rs12732658 | - | *MEF2D* | **0.046** | *IQGAP3* | 0.55 |
| rs12747719 | - | *MEF2D* | 0.180 | *IQGAP3* | 0.87 |

^*^Data were not available (N/A) from GTEx

**Supplementary Table 5. PCR primers.**

| **Primer** | **Primer sequence** | | | |
| --- | --- | --- | --- | --- |
| *Real-time PCR* |  | | | |
| *ZNF100* 5’ qPCR | TAGCCTGTGTGGCCCTCTG | | | |
| *ZNF100* 3’ qPCR | GGCCACATCCCTAAACGTCA | | | |
| *MEF2D* 5’ qPCR | TTAGATCTGAACAATGCCCAGC | | | |
| *MEF2D* 3’ qPCR | AGGCTGGTAAGGAGGAGAGC | | | |
| *ACTB* 5’ qPCR | GGAAATCGTGCGTGACATTAAGG | | | |
| *ACTB* 3’ qPCR | AGTACTTGCGCTCAGGAGGAGC | | | |
| *ZNF100 3C* |  | | | |
| 3C Bait | GCCATTCCTAACTCCCAGTTCCAACAATAGG | | | |
| 3C Fragment 1 | GTTCATATCACCTTCTATCATTAGGGCCCAGTTCC | | | |
| 3C Fragment 2 | AACCTCAGGTGTGCACCACCACACC | | | |
| 3C Fragment 3 | TGCTATTTGTGATTGGTGGCCATTTAAGACC | | | |
| 3C Fragment 4 | TGTTGTGTCTTCTGAGGTTATCACCTGAAGGG | | | |
| 3C Fragment 5 | GCAAGACTCCAAAGTGGGCCAGACC | | | |
| 3C Fragment 6 | TCCACCCACAACAATAAACAGAAGCTGTGG | | | |
| 3C Fragment 7 | AAATGATAAGGACAGCCAACCGGAAGGC | | | |
| 3C Fragment 8 | CCAATTTGTCTGATGTTCTGGCACTTTGC | | | |
| 3C Fragment 9 | GGCAAAGCCTTCAACTGGTTCTCAACC | | | |
| 3CFragment 10 | ATTGAACAAAGTTTGAGCAACTGCTTCAGAGG | | | |
| 3C Fragment 11 | TGAGTGTCTTTCATACCATTACCATCAGCACC | | | |
| 3C Fragment 12 | CCTTCCCACCAAAGCAAGCCATGG | | | |
| 3C Fragment 13 | TGGAGGACTAATTAACGCAGACTCCATCATAAGG | | | |
| 3C Fragment 14 | AGGCAGTTTAAGTGGGTAAAAGAGTTCTCACTGG | | | |
| 3C Fragment 15 | CCAGATGAAATGGCTGCACTGTCTGG | | | |
| 3C Fragment 16 | TAACAGAGAGAGAGAAAGCTCCTCATGC | | | |
| 3C Fragment 17 | ACCAGCCAGGTAAAGCCACGTGAGC | | | |
| 3C Fragment 18 | TCCAGTGGCCTTCATCACATGCTGG | | | |
| 3C Fragment 19 | GCTGTCACCATGACATCTGCACTCATGG | | | |
| 3C Fragment 20 | TTGTAGCCTTGCACCACATAAAGAGAAACTGC | | | |
| *Allele-specific 3C* |  | | | |
| Reverse primer | GGTAAGAAGGTGGTGCTGACACATT | | | |
| Sequencing | GCTTCTCATTAAGATCACATGACCAGTTGG | | | |
| *MEF2D cloning and sequencing* | | | | |
| *MEF2D* promoter 5’ | GGTACCACAGGTAGAGGGAGCAACTGAGC | | | |
| *MEF2D* promoter 3’ | AAGCTTAGCAAAGAGGGACACAGGATCC | | | |
| *MEF2D* prom seqfwd1 | TACTTAGACTCCCACCTCCCAA | | |  |
| *MEF2D* prom seqfwd2 | AAGCTAGGCTGAGCTGTGCCTA | | |  |
| *MEF2D* prom seqfwd3 | GGGATTACCTAGGGGACTGTTT | | |  |
| *MEF2D* PRE1 5’ | ACCGGTCTTGAGAGAGAGCCTTTTGCTATCC | | | |
| *MEF2D* PRE1 3’ | GTCGACATCAATCTTGGAGGAAGGTCGAG | | | |
| rs942964 T 5’ | GTAATTTTGGCAGCAC**T**TCATGAACATTGCTG | | | |
| rs942964 T 3’ | CAGCAATGTTCATGA**A**GTGCTGCCAAAATTAC | | | |
| rs11264489 C 5’ | CCTGTGTCTTC**C**CGGCAACGCAG | | | |
| rs11264489 C 3’ | CTGCGTTGCCG**G**GAAGACACAGG | | | |
| rs10159180 G 5’ | CTTGATCGTTACCTC**G**TTCAAACTCCAGTCC | | | |
| rs10159180 G 3’ | GGACTGGAGTTTGAA**C**GAGGTAACGATCAAG | | | |
| rs11264488 C 5’ | CAATAATAAAA**C**AGAAGAAAAATAACGGCATACAG | | | |
| rs11264488 C 3’ | CTTCTGTTTTATTATTGTTTTTT**G**GTGGACTGGAG | | | |
| *MEF2D* PRE1 seqfwd1 | CTGGAGTGCAGTGGCGCAAT | |  |  |
| *MEF2D* PRE1 seqfwd2 | CCTGGCATAAAGCAGGAATGAAA | |  |  |
| *MEF2D* PRE1 seqfwd3 | GGCAATGGAGGAAGTGACAGAA | |  |  |
| *MEF2D* PRE2 AgeI5’ | ACCGGTCTCTTGTCTGTGGTCTGTTTAATATGTGG | | | |
| *MEF2D* PRE2 SalI3’ | GTCGACAATTGTCCTCCAAAGAGGTTACACTGG | | | |
| rs7550381 T 5’ | CTCTTCCTTCCCC**T**GGGGGACAGAGAG | | | |
| rs7550381 T 3’ | CTCTCTGTCCCCC**A**GGGGAAGGAAGAG | | | |
| rs947661 T 5’ | GC**T**ACTCTAATTTCTACCCCACCCGTTTTACACAC | | | |
| rs947661 T 3’ | GTGGGGTAGAAATTAGAGTAGCTTCTCTTCTC**A^#^**GC | | | |
| *MEF2D* PRE2 seqfwd1 | | TTTCTGTAGAGCCGGCCTTT |  |  |
| *MEF2D* PRE2 seqfwd2 | | AGACACCTGCCCTGACATCTGAA |  |  |
| *ZNF100 cloning and sequencing primers* | | | | |
| *ZNF100* promoter 5’ | GGTACCACAATGTGCCTGGTCTGAAATGC | | | |
| *ZNF100* promoter 3’ | AAGCTTAGCAGAAGACACAGAGAAGTGAGAGC | | | |
| rs4467196 T 5’ | | ATCCACATGTGGCT**T**ACATTCATTTTAGATG | | |
| rs4467196 T 3’ | | CATCTAAAATGAATGT**A**AGCCACATGTGGAT | | |
| *ZNF100* PRE1 5’ | | ACCGGTTGCTGGGCCCTTTATCTAAATCC | | |
| *ZNF100* PRE1 3’ | | GTCGACAATGTGTCAGCACCACCTTCTTACC | | |
| rs10421113 A 5’ | | GCACCTGAGAC**A**GGAAAGGAGAAAC | | |
| rs10421113 A 3’ | | GTTTCTCCTTTCC**T**GTCTCAGGTGC | | |
| rs10421342 A 5’ | | CACCTTTGCACTA**A**AGGGTGGTTACGATACC | | |
| rs10421342 A 3’ | | GGTATCGTAACCACCCT**T**TAGTGCAAAGGTG | | |
| rs4808295 T 5’ | | GAAACACCCA**T**TCATGAACCCTTTCC | | |
| rs4808295 T 3’ | | GGAAAGGGTTCATG**A**ATGGGTGTTTC | | |
| rs78985149 G 5’ | | CTCTTGAGCACAT**G**GTACCTGCTTAATAATTATTG | | |
| rs78985149 G 3’ | | CAATAATTATTAAGCAGGTAC**C**ATGTGCTCAAGAG | | |
| *ZNF100* PRE1 seqfwd1 | | GTGGGACCAACATTACCAAGTGATT | | |
| *ZNF100* PRE2 5’ | | ACCGGTGTAGAGACAGGGTTTCG | | |
| *ZNF100* PRE2 3’ | | GTCGACGCCCAGTGTTAAGTACG | | |
| rs11879157 A 5’ | | CCTAACGGCC**A**TCTTCATTTACATTCAAACC | | |
| rs11879157 A 3’ | | GGTTTGAATGTAAATGAAGA**T**GGCCGTTAGG | | |
| rs4547465 T 5’ | | CTGTTGATTCTATATTTTGG**T**TATTGTGAAGAGTG | | |
| rs4547465 T 3’ | | CACTCTTCACAATA**A**CCAAAATATAGAATCAACAG | | |

Underlined sequence indicates restriction enzyme site.

**Bolded base** indicates SNP locus.

**Supplementary Table 6. All studies included in the meta-analysis according to chemotherapy subset (“Any chemo” & “Standard chemo”)**

| **Study (Location)** | **Ascertainment** | **Follow-up** | **^a^Overall Survival** | | **^a^Progression-Free Survival** | |
| --- | --- | --- | --- | --- | --- | --- |
|  |  |  | **Any Chemo (N)** | **Std Chemo (N)** | **Any Chemo (N)** | **Std Chemo (N)** |
| AUS (Australia) | Patients were diagnosed from 2002-2006; recruited through surgical treatment centers throughout Australia & cancer registries of Queensland, S. Australia, W. Australia, Tasmania, New South Wales & Victoria | Medical records reviewed at 6 - 12 month intervals | 976 | 584 | 976 | 584 |
| ^b^BAV (Southeast Germany) | Patients recruited through hospitals in Erlangen, Northern Bavaria, Germany from May 2002 to August 2008 | Cancer registry, medical records, and patient contact | 57 | 29 | 0 | 0 |
| BEL (Belgium) | Patients attending the Gynecologic Oncology Unit at the Leuven University Hospital diagnosed with incident ovarian cancer from 2009 onwards | Patient contact and vital statistics | 272 | 123 | 272 | 123 |
| BVU (USA) | Patients diagnosed or treated at Vanderbilt University Medical Center clinics. Includes all ovarian cancer patients included in the Tumor Registry, as well as patients not included in the Tumor Registry but with verified diagnoses. | Data were abstracted from study participants clinical electronic medical record | 64 | 41 | 64 | 41 |
| CNI (Spain) | Patients ascertained through CNIO familial cancer consultancy or referrals to CNIO for BRCA1/2 mutation screening or attending hospitals in Madrid in Medical Oncology Divisions; | Data obtained from medical records by research nurses and data managers at centres | 25 | 15 | 25 | 15 |
| HAW (USA: Hawaii & Southern CA) | Hawaii Tumor Registry; patients diagnosed between 1993 and 2008 | Standard US NCI SEER-registry follow-up methods and review of the medical charts | 50 | 16 | 50 | 16 |
| ^b^HJO (Germany) | Patients ascertained from the Hannover Medical School or the Friedrich-Schiller University Jena | Clinical and pathology data were drawn from medical records. | 78 | 24 | 0 | 0 |
| HOP (USA: OH, PA and NY) | Patients were identified from three catchment areas (western PN, northern OH & western NY) through a variety of sources including physician offices, cancer registries and pathology databases. | Structured interview using detailed questionnaires, and review of medical records from physicians, pathologists, hospitals. | 436 | 197 | 436 | 197 |
| HSK (Germany) | Patients attending the Gynecologic Oncology Unit at the Dr. Horst-Schmidt Kliniken, Wiesbaden, diagnosed with incident ovarian cancer between 2005-2009 | Institutional database, medical records and patients contact at least annually | 151 | 86 | 151 | 86 |
| ICN (multi-center) | Patients were enrolled in ICON7 between 2006 and 2009 at 263 centers in the United Kingdom, Germany, France, Canada, Australia,New Zealand, Denmark, Finland, Norway,Sweden, and Spain; and were randomly assigned in a 1:1 ratio to receive carboplatin- paclitaxel (standard-chemotherapy group), or to the same regimen plus bevacizumab | Patients were follow-up every 3 months until disease progression was documented. If no evidence of disease progression, follow-up interval was extended to every 6 months during years four and five and yearly thereafter. After documented disease progression, patients were followed every 6 months up to 5 years after study entry, and yearly thereafter to document survival and ovarian cancer therapy | 363 | 162 | 366 | 164 |
| LAX (USA: Southern CA) | Patients were identified through the Women's Cancer Research Institute biorepository from 1989 onwards. Patients presenting to the gynecologic cancer service with epithelial ovarian cancer are identified in IRB 901 (Tissue Bank) and IRBs 1080 and 4049 (Gilda Radner Hereditary Cancer). | Annual chart abstraction and cancer registry updates | 221 | 95 | 221 | 95 |
| MAC (USA: North Central) | Patients attending Mayo Clinic diagnosed from 2000 onwards identified outside a six state surrounding region or greater than one year from diagnosis. | Patient contact and vital statistics | 138 | 63 | 138 | 63 |
| MAL (Denmark) | Incident cases diagnosed 1994 -1999 from municipalities of Copenhagen & Frederiksberg & surrounding counties. | Danish Civil Registration System and Danish Register of Causes of Death | 376 | 112 | 376 | 112 |
| MAY (USA: North Central) | Patients attending Mayo Clinic enrolled within one year of diagnosis from 2000 onwards identified in a six state surrounding region. | Patient contact and vital statistics | 1005 | 394 | 1005 | 394 |
| ^b^NCO (USA: Central and Eastern NC) | Patients were identified through the North Carolina Central Cancer Registry by using rapid case ascertainment in a 48 county region of NC. Pathology reports for ovarian cancer patients were forwarded to the Central Cancer Registry and then to the study office within 2 months of diagnosis. | National Death Index and North Carolina Central Cancer Registry every 18-24 months | 212 | 121 | 0 | 0 |
| NEC (USA: NH & Eastern MA) | Patients were identified through state-wide cancer registries and hospital tumor boards in eastern Massachusetts and New Hampshire. | Annual medical record abstraction and death record database updates | 66 | 30 | 66 | 30 |
| NOR (Norway) | All patients treated for suspected gynecologic cancer from one region of Norway | Data was collected by reviewing the medical records in the hospital | 168 | 98 | 0 | 0 |
| OPL (Australia) | Women aged 18-79 years with primary invasive epithelial ovarian cancer diagnosed between May 2012 and October 2014 identified through the major treatment centres in each state in Australia. | Medical records reviewed at recruitment and then annually. | 384 | 96 | 330 | 96 |
| ORE (USA: OR) | Registry and tissue repository of patients with ovarian cancer (or at risk for ovarian cancer and control case undergoing surgery for benign gynecologic conditions that aren't included in OCAC). Focus of the current research is on Fanconi DNA repair genes and proteins. | Cancer registry and electronic medical record reviews every three months | 14 | 0 | 14 | 0 |
| PVD (Denmark) | All patients admitted with a pelvic mass at Rigshospitalet, University of Copenhagen, are included in the study with a blood sample less than 14 days before surgery/diagnosis of ovarian cancer and with FFPE and fresh frozen tissues if possible | Patient contact, recorded in online database and vital statistics | 180 | 0 | 180 | 0 |
| RBH (Australia) | Patients treated at Royal Brisbane Hospital diagnosed from 1985-1996 and recruited to the Biospecimen Bank in the Department of Obstetrics and Gynecology | Medical record abstraction | 116 | 0 | 116 | 0 |
| SRO (UK) | Patients randomised into a prospective phase III comparison of paclitaxel-carboplatin versus docetaxel-carboplatin as first line chemotherapy in stage Ic-IV epithelial ovarian cancer (SCOTROC 1) which recruited from 1998 to 2000 | Performed every 2 months until progressive disease was documented. If no evidence of progression within 2 years of randomization, follow-up interval extended to 3 months during the 3rd year, and to 4 months for the 4th year. Thereafter (or after progression), patients were followed every 6 months. | 124 | 49 | 124 | 49 |
| ^c^TCGA (UK & North American) | Patients diagnosed with serous tumours from 2006 at 15 participating UK and North America cancer centers | Patients followed according to site protocol. Clinical data finalized in August 2010. More information can be obtained at http://cancergenome.nih.gov/ | 335 | 145 | 337 | 147 |
| UHN (Canada) | All women attending the Division of Gynecologic Oncology outpatient clinics at Princess Margaret Hospital who have consented to provide a blood sample to the GYNE Site Biobank, and have a confirmed diagnosis of epithelial ovarian cancer. | Clinical data obtained via medical record | 131 | 53 | 131 | 53 |
| VAN (Canada) | Patients attending VGH and/or BC Cancer Agency Division of Gynecologic Oncology | Clinical data obtained via medical record review | 143 | 52 | 143 | 52 |
| WMH (Australia) | Patients treated at Westmead Hospital, Sydney, diagnosed from 1992 onwards and recruited to the Gynaecological Oncology Biospecimen Bank at Westmead (GynBiobank) | Medical record abstraction | 75 | 35 | 75 | 35 |
| **Total** |  |  | **6160** | **2620** | **5596** | **2352** |

^a^N is the largest dataset with SNP data and complete covariate data for all analyzed SNPs; differences in N for specific SNP analyses may be due to a small number of missing genotypes.

^b^These sites were excluded from PFS analysis due to missing or inconsistent data

^c^Clinical follow-up, chemotherapy and genotype data downloaded from <http://cancergenome.nih.gov/>
